# Deficits in Implicit Attention to Social Signals in Schizophrenia and High Risk Groups: Behavioural Evidence from a New Illusion

**DOI:** 10.1371/journal.pone.0005581

**Published:** 2009-05-15

**Authors:** Mascha van 't Wout, Sophie van Rijn, Tjeerd Jellema, René S. Kahn, André Aleman

**Affiliations:** 1 Department of Experimental Psychology, Helmholtz Institute, Utrecht University, Utrecht, The Netherlands; 2 Department of Psychology, Brown University, Providence, Rhode Island, United States of America; 3 Department of Clinical Child and Adolescent Studies, Centre for the Study of Developmental Disorders, Leiden University, Leiden, The Netherlands; 4 Department of Psychology, University of Hull, Hull, United Kingdom; 5 Department of Psychiatry, Rudolf Magnus Institute of Neuroscience, University Medical Center Utrecht, Utrecht, The Netherlands; 6 BCN Neuroimaging Center, University of Groningen, Groningen, The Netherlands; James Cook University, Australia

## Abstract

**Background:**

An increasing body of evidence suggests that the apparent social impairments observed in schizophrenia may arise from deficits in social cognitive processing capacities. The ability to process basic social cues, such as gaze direction and biological motion, effortlessly and implicitly is thought to be a prerequisite for establishing successful social interactions and for construing a sense of “social intuition.” However, studies that address the ability to effortlessly process basic social cues in schizophrenia are lacking. Because social cognitive processing deficits may be part of the genetic vulnerability for schizophrenia, we also investigated two groups that have been shown to be at increased risk of developing schizophrenia-spectrum pathology: first-degree relatives of schizophrenia patients and men with Klinefelter syndrome (47,XXY).

**Results:**

We compared 28 patients with schizophrenia, 29 siblings of patients with schizophrenia, and 29 individuals with Klinefelter syndrome with 46 matched healthy control subjects on a new paradigm. This paradigm measures one's susceptibility for a bias in distance estimation between two agents that is induced by the implicit processing of gaze direction and biological motion conveyed by these agents. Compared to control subjects, patients with schizophrenia, as well as siblings of patients and Klinefelter men, showed a lack of influence of social cues on their distance judgments.

**Conclusions:**

We suggest that the insensitivity for social cues is a cognitive aspect of schizophrenia that may be seen as an endophenotype as it appears to be present both in relatives who are at increased genetic risk and in a genetic disorder at risk for schizophrenia-spectrum psychopathology. These social cue–processing deficits could contribute, in part, to the difficulties in higher order social cognitive tasks and, hence, to decreased social competence that has been observed in these groups.

## Introduction

One of the cardinal dysfunctions associated with the schizophrenia phenotype concerns disturbances in social functioning [1]. Although some researchers have argued that this might be a consequence of severe psychopathology, others have demonstrated that social dysfunction is relatively independent of symptomatology [2]. This latter view is further supported by findings that disturbances in social functioning are already present in early adolescence and often precede the onset of psychosis [3]–[5]. In the search for determinants of social dysfunction in schizophrenia, adequate cognitive processing of social information appears to be of crucial importance. In the last decade a growing body of research demonstrated deficits in social information processing in schizophrenia [6], including difficulties in emotion recognition [7]–[9], an inability to understand and manipulate other people's behaviour in terms of their mental states, also called Theory of Mind, as well as an insensitivity to interpersonal social cues that refer to someone's affect and goals [10]. Interestingly, these social cue processing deficits seem to be independent of intelligence, i.e. not attributable to a generalized performance deficit [11], but are related to negative symptoms of schizophrenia, such as emotional withdrawal [12] and skills to perceive, process, and send social signs [13].

Indeed, the ability to quickly and effortlessly process social cues is an important underlying characteristic of successful social interactions and communication [14] as it allows a continuous interpretation of rapidly changing social signals. Examples of such basic social signs that are processed fast and effortlessly, or implicitly, are gaze direction, head orientation and body postures [15]. These cues can give clues about someone's intentions, goals and beliefs [16]. This fast and effortless processing of social cues may be especially relevant for construing a sense of ‘social intuition’ in which involuntary and implicit processes are crucial [17]. Intuitions have been described as follows: “intuitions are fast and take into account non-consciously generated information, gathered from experience, about the probabilistic structure of the cues and variables relevant to one's judgments, decisions, and behaviour” [18]. Although schizophrenia patients seem to fail in areas of social intuition and the implicit processing of social cues in social interactions as observed in their social behaviour [19], studies that address the ability to effortlessly process basic social cues in schizophrenia are scarce. The present study sought to remedy this, and examined the influence of the implicit processing of social cues on a distance judgment task in schizophrenia.

In addition to patients with schizophrenia and healthy controls, we included two other groups in the study: a) individuals at increased genetic risk for schizophrenia, i.e. siblings of schizophrenia patients and b) individuals with an X chromosomal disorder who are at risk for developing schizophrenia-like psychopathology, i.e. men with Klinefelter syndrome. Biological siblings of patients with schizophrenia have been shown to be at significantly higher risk for the development of schizophrenia [20], and display cognitive deficits similar to those observed in schizophrenia patients, although to a lesser degree [21]. Inclusion of the sibling group allowed us to study social cognitive deficits that are related to a genetic vulnerability to schizophrenia, without confounding environmental influences as hospitalization, medication and psychopathology. Support for the role of genetic mechanisms in social cognitive deficits comes from studies demonstrating abnormalities in the processing of social-emotional cues in biological relatives of patients with schizophrenia [22], [23]. This fits with the finding that social skills are under considerable genetic control in the general population.

The third experimental group consisted of men with Klinefelter syndrome who have an extra X-chromosome (47,XXY chromosomal pattern). Klinefelter syndrome has been associated with serious social difficulties and social cognitive deficits, such as high levels of social anxiety, communication difficulties and impaired social skills as well as deficits in interpreting non-verbal social signals [24]–[26]. Furthermore, high levels of schizotypal traits and schizophrenia symptoms have been observed in men with Klinefelter syndrome and include ideas of reference, unusual perceptual experiences, magical thinking, odd speech, disorganized thinking, suspiciousness and excessive social anxiety [27]–[30]. In addition, the life-time prevalence of psychotic disorders in XXY men appears to be 16 times higher as compared to men from the general population [30] and Klinefelter has been associated with an increased relative risk of being hospitalized with severe psychopathology such as schizophrenia spectrum pathology [28]. Moreover, the prevalence of the XXY chromosomal pattern is higher among men with schizophrenia. Taken together this has led others to propose that Klinefelter syndrome may serve as a genetic model for psychosis [29], [31]. Therefore, similar to relatives of schizophrenia patients, this genetic population can be considered a high risk population for the development of schizophrenia. Considering the social cognitive deficits, Klinefelter syndrome may specifically serve as a model for investigating the contribution of social perception impairments to schizophrenia symptoms.

An additional advantage arising from studying Klinefelter men is knowledge about the precise genetic aetiology of this syndrome, in contrast to the limited knowledge of the genetic underpinnings of social cognitive impairments in schizophrenia. It has been hypothesized that the X chromosome may harbour genes that are crucially involved in development of the social brain [32]. Similarities between patients with schizophrenia, their siblings and XXY men might point to a role of the X-chromosome in the development of cognitive systems that are important for processing basic social signals [33] and because social perception deficits in Klinefelter syndrome may result from an X chromosomal abnormality, this may have heuristic value in the search for the genetic mechanisms underlying social perception deficits in schizophrenia. Indeed, there is reason to suspect the involvement of sex chromosomes as it might explain, at least in part, the sex differences that have been observed in social cognitive skills in the general population as well as in schizophrenia populations [34].

The aim of the present study was to investigate the implicit processing of basic social cues in three groups on the schizophrenia continuum, i.e. in individuals with schizophrenia, individuals with an increased genetic risk for schizophrenia and individuals with a genetic disorder who show schizophrenia-like symptoms. To this end we used a new paradigm involving a bias in the judgment of the distance between two agents induced by the implicit, i.e. effortless processing of social cues conveyed by these agents. In this task the social cues consisted of the direction of attention (gaze direction) and implied biological motion (body postures). We choose these social cues based upon an extensive body of research showing that biological motion can be accurately and effortlessly perceived [35]–[39] and that direct gaze serves as a precursor to social interaction [40]–[42]. Hence, these social cues can induce the sensation of people (dis-)engaging in a social interaction when their gaze or body postures attend towards (or away from) each other. Consequently, the automatic or implicit processing of gaze direction and implied biological motion can result in people judging the agents as closer together, compared to reference objects, whilst objectively this is not the case (see Jellema *et al.*, 2004 for published pilot data in form of an abstract).

We hypothesized that patients with schizophrenia would demonstrate difficulties in the effortless or implicit processing of social cues compared to control participants, i.e. patients may show no response bias congruent with the direction of the social cues whereas this would be the case in control participants. We expected a similar lack of response bias in siblings of patients with schizophrenia and XXY men albeit to a lesser extent compared to patients with schizophrenia. Furthermore, we investigated the relationship between schizophrenia symptomatology and social cue processing in patients with schizophrenia. We predicted that lack of social cue processing would be especially prevalent in patients with negative symptoms, since these patients are in particular characterized by social-emotional disturbances.

## Materials and Methods

### Ethics Statement

The local ethics committee, METC-UMCU approved the study and all subjects provided written informed consent after the procedure had been fully explained according to Declaration of Helsinki.

### Participants

33 Patients (23 men, 10 women) with a diagnosis of schizophrenia were recruited at the University Medical Centre Utrecht. All patients met the DSM-IV criteria for schizophrenia, as confirmed by the Comprehensive Assessment of Symptoms and History interview (CASH) [43] semi-structured interview designed for research in the major psychoses and was administered by a psychiatrist. Patients were also screened for affective disorders, i.e. depression and mania, and substance-related disorders, with the CASH. Most patients were diagnosed with paranoid schizophrenia (n = 22), one with disorganized type, one with residual type, six with undifferentiated type and three with schizophreniform disorder. Patients were clinically stable; four patients were inpatients and in remission and 29 were outpatients. 31 Patients received medication (30 patients only antipsychotics, such as leponex (n = 13), quetiapine (n = 4), olanzapine (n = 6), risperidone (n = 8) and one patient also received oxazepam). Symptoms and severity were independently rated by two raters with the Positive and Negative Syndrome Scale (PANSS) [44]. Raters were trained by a qualified trainer and followed inter-rater reliability training every six months. Mean positive symptoms was 14.22 (SD 5.22, range 7–27), negative symptoms 14.84 (SD 5.78, range 7–29) and general psychopathology 26.66 (SD 6.84, range 17–47). Mean duration of illness was 9.44 years (SD 8.01) and mean age of onset was 23.83 years (SD 5.45).

32 Siblings of patients with schizophrenia (12 men, 20 women) were recruited through advertisements at the Ypsilon website, which is a website dedicated to relatives of patients with schizophrenia. The diagnosis of schizophrenia for the affected sibling was confirmed with a CASH interview. However, due to ethical reasons we were unable to verify the diagnosis of schizophrenia for 12 affected siblings with the CASH interview.

32 Men with Klinefelter syndrome (47,XXY) were studied. The participants were recruited from the Dutch Klinefelter Association, and were not selected for psychological, behavioural or cognitive abnormalities. Diagnosis of Klinefelter syndrome was confirmed by karyotyping, using standard procedures. 50 Non-psychiatric control participants (31 men, 19 women) were drawn from the general population via advertisements in local newspapers.

Inclusion criteria for all participants were age between 18 and 65 years and good physical health. Exclusion criteria were neurological conditions, history of head injury with loss of consciousness, recent history of alcohol and substance abuse, or mental retardation. None of the control participants and siblings had a history of psychiatric illness or use of psychiatric medication confirmed with the Mini International Neuropsychiatric Interview plus [45]. The Dutch translation of the National Adult Reading Test (NART) [46] and Raven's Advanced Progressive Matrices [47] were used to match the groups on estimates of verbal and performance intelligence level, respectively [48]. See Table 1 for demographic data of participants that were included in the analyses as some participants were excluded from the analyses due to attentional problems (see also methods social distance judgment task and statistical analyses).

**Table 1:** Demographic data (mean (SD)) of participants included in the Social Distance Judgment Task analysis.

Variable	Patients	Siblings	Klinefelter men	Controls	P^1^	P^2^
N	28	29	29	46		
Age in years	32.4 (7.5)	34.6 (10.7)	38.1 (8.5)	31.9 (9.2)	0.45	0.08
Male∶female ratio	18∶10	11∶18	1∶0	27∶20	0.11	NA
Education in years	14.3 (2.8)	16.2 (1.9)	13.9 (2.7)	14.9 (2.6)	0.01	0.56
Parental education in years	13.9 (2.9)	14.6 (2.7)	NA	13.2 (2.9)	0.27	NA
NART	103.6 (8.2)	104.5 (8.1)	102.7 (8.6)	107.6 (9.5)	0.13	0.09
Raven's Matrices	NA	109.2 (9.9)	107.7 (14.4)	108.4 (13.8)	0.79	0.24

P^1^: Between-group comparisons of patients with schizophrenia, siblings of patients and control participants with ANOVA, except male∶female ratio is analyzed with non-parametric Kruskal Wallis test, df = 100; P^2^: Between-group comparisons of Klinefelter men and male controls with Student's t-test, df = 52; NA = Not available.

### Social Distance Judgment Task

The Social Distance Judgment Task measures the illusion of de- or increasing distance caused by the implicit, or effortless, processing of social cues. The underlying principle behind the task is that the perceived distance between two agents will be influenced by social cues conveyed by these agents in comparison to the perceived distance between two geometrical objects that do not signal social intentions, even though the actual distance between the two agents and the two geometrical objects is the same. The social cues signalled by the two agents will result in a response bias paralleling the strength of social cues, i.e. the more social cues are present the stronger the bias will be. This had been confirmed previously in a pilot study (published abstract Jellema *et al.*, 2004).

**Figure 1:**
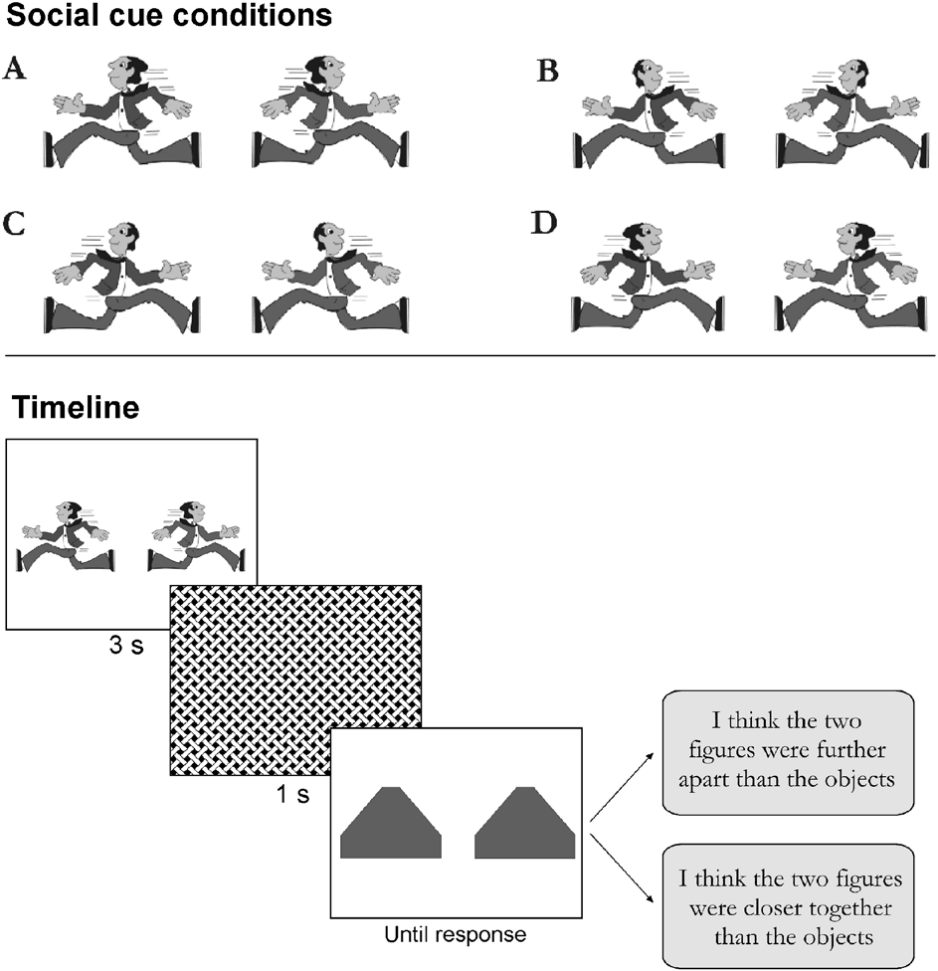
Response to social cues. Top panel: Left to right: increasing strength of social cues leading to the response: ‘Cartoons Closer’. Bottom panel: Example of a single trial.

Stimuli were pairs of cartoon figures shown in running postures conveying two different social cues: gaze direction (figures looking away or towards each other) and biological motion (figures running away or towards each other). Head and body of the cartoon figures were pointing in the same direction, or in opposite directions, amounting to a total of four different compositions of cartoon figures, see Figure 1, top panel. One (male) cartoon figure was used, selected from the CorelDraw graphical package. Cartoon figures were always presented in pairs as each other's mirror-image (as displayed in Figure 1, bottom panel). All faces had the same, fairly neutral, expression.

A pair of cartoon figures was presented for 3 s, after which a mask of 1 s was shown, followed by a pair of geometrical figures (see Figure 1, bottom panel, for an example of a trial). Participants had to choose one of two possible responses: (1) ‘I think the two cartoon figures were closer together than the two geometrical objects’ and (2) ‘I think the two cartoon figures were further away from each other than the two geometrical objects’. For convenience, we labelled response 1 as ‘Cartoons Closer’ and response 2 as ‘Cartoons Farther’. We chose to use this forced-choice paradigm to increase the likelihood to detect a response bias.

The task consisted out of 30 trials evenly distributed over the four social cue levels and so-called catch-trials, resulting in every social cue level occurring six times. Except for the six catch-trials, the distance between the geometrical figures was always the same as the distance between the cartoon figures. These catch-trials were used to allow exclusion of those participants from analysis that did not pay proper attention to the task. Participants who made more than two errors in the catch-trials were excluded from the analyses. Three different distances of 2, 3 or 4 cm between cartoons and geometrical figures were randomly presented. In the catch-trials there was a 2 cm difference between the geometrical figures and the cartoon figures. Before the onset of the task, participants completed six practice trials.

The maximal height and width of the geometrical objects matched those of the corresponding cartoon figures. The dimensions on the screen were 4.8×6.5 cm (height×width) for each cartoon figure. The figures had been digitally adapted such that the mass distribution on either side of the vertical midline was identical, with the eye positioned exactly at the midline of the figure and centred in the head. However, the wind caused the jacket and tie to adopt a sideway position resulting in a slight asymmetry in mass distribution. Although this slight asymmetry in mass distribution is unrelated to this study, it is important to briefly mention as it resulted in an independent low-level illusion (also observed in pilot data: Jellema *et al.*, 2004). This low-level illusion is attributable to a less massive appearance due to spaces in the running cartoons than the corresponding pyramidal-shaped geometrical objects. Therefore the least massive objects will be judged furthest away from the observer, and thus inferred to be furthest away from each other. For that reason we expect that, when participants are not influenced by social cues in their distance judgments, a large bias toward the “Cartoons Farther” response (roughly 75% of responses, and 25% “Cartoons Closer”) instead of expecting participants to respond randomly, i.e. choosing 50% “Cartoons Closer” and 50% “Cartoons Father” on the task. Given that this response bias occurs irrespective of social cues this effect is called low-level.

### Statistical Analysis

Data from the Social Distance Judgment Task was analyzed with General Linear Model repeated measures ANOVA of within subject contrast with increasing social cue strength as a within subjects variable (four strength levels A to D, see Figure 1, top panel). The order of social cue conditions was based on pilot data demonstrating that the biological motion cue had a stronger effect on distance judgment than the gaze direction cue (published abstract Jellema et al., 2004). Our pilot data further revealed that the gaze direction cue facilitated the motion cue (or the other way around) to have an effect, i.e. both social cues are required to get a stronger visual illusion. Given the incongruency of biological motion and gaze direction in condition B and C (see Figure 1, top panel), we thus did not expect a significant difference in distance judgments between these conditions. Repeating the analyses with only three levels of social cues strength (A, BC, D) did not alter the results described below.

The repeated measures ANOVA was first done separately for the different groups (controls, patients, siblings, Klinefelter men) to investigate whether there was a significant linear increase consistent with social cue strength in each of the groups. Second a similar GLM repeated measures of within subject contrast with the four social cue strength levels as a within subjects variables, but with Group (control vs. experimental groups: patients, siblings) as between subject factor tested for differences between the groups on the influence of social cues on distance judgment. Because only males are affected with Klinefelter syndrome, we performed a separate GLM repeated measures analysis in which the between subjects factor Group consisted out of Klinefelter men versus male controls only.

Five patients with schizophrenia, three siblings, three Klinefelter men and four control participants made more than two errors in the catch-trials and were not included in further analyses. See Table 1 for demographic data of participants included in the analyses.

## Results

Across all groups we observed the presence of a low-level effect that is noticeable in the general large bias toward the “Cartoons Farther” response compared to the “Cartoons Closer” response across all conditions and irrespective of social cues.

### Social Distance Judgment Task: Control Subjects, Patients, and Siblings

First to determine whether we found an effect of social cues on distance judgments we examined performance in the *control group* using a repeated measure ANOVA with the four social cues as within-subjects factors. This analysis revealed a significant main effect of the different levels of social cues (F(3,43) = 7.42, p = 0.006, indicating that the different social cues had different effects on the distance judgments in the task. A post hoc t-test confirmed that the two extreme conditions, i.e. condition A vs. D (see Figure 1, top panel for a reference to the different conditions), differed significantly from each other in distance estimations, t(45) = −3.63, p = 0.001. Therefore, we tested whether there was a significant *linear increase* in the percentage of response ‘Cartoons Closer’ with increasing social cue strength and this was indeed what we found, F(1,45) = 14.27, p = 0.0005. This shows that there was an influence of social cue strength on distance judgments according to the social cues.

We repeated the same analyses for the *patient* group, but did not observe a significant main effect of a linear increase in the percentage of the response ‘Cartoons Closer’ with increasing social cue strength, F(1,27) = 0.34, p = 0.56. This shows that the percentage response ‘Cartoons Closer’ did not change with increasing social cue strength in the patient group. Post hoc t-test confirmed a lack of significant difference between the congruent social cue conditions A vs. D in the patient group, t(27) = 0.43, p = 0.67, and in the incongruent social cue conditions B vs. C, t(27) = 0.39, p = 0.699.

Remarkably, this absence of a significant main effect of a linear increase according to social cue strengths, and thus the suggested absence of a response bias, was also found in the *sibling* group, F(1,28) = 0.77, p = 0.39. Again post hoc t-tests did not show a significant difference between social cue conditions A vs. D, t(28) = −0.77, p = 0.45, or between social cue conditions B vs. C, t(28) = −0.59, p = 0.56.

**Figure 2:**
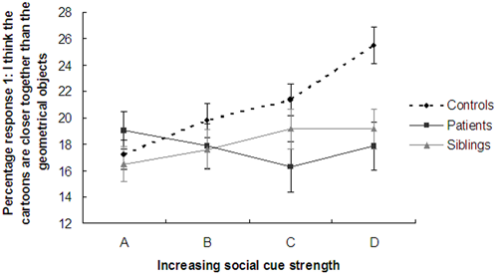
Linear increase in response 1 (“Cartoons Closer”) consistent with social cue strength in healthy control subjects, but not in patients or sibling of patients.

To test whether the pattern on the task was different for the three groups we performed a repeated measure ANOVA with the four social cues as within-subjects factors and group (patients, siblings and controls) as between-subject factor. There was a significant main effect of a linear response due to increasing social cue strength, F(1,100) = 4.33, p = 0.04. In addition, we observed a significant interaction between the groups and levels of social cue strength, F(2,100) = 3.79, p = 0.026, demonstrating that the pattern of the response ‘Cartoons Closer’ in proportion to social cue strengths differed between patients with schizophrenia, siblings and control subjects. See Figure 2.

Indeed, post-hoc tests revealed that the control group differed significantly from the patient group in sensitivity for social cues (F(1,72) = 8.06, p = 0.006), which was specifically due to a significant difference between social cue condition A vs. D, t(73) = −2.66, p = 0.01. The sibling group did not differ from the control group (F(1,73) = 2.21, p = 0.14), nor from the patient group (F(1,55) = 1.09, p = 0.30).

Previous studies demonstrated sex differences in social-emotional information processing in schizophrenia. Although there was no significant difference between males and females in the control (F(1,44) = 0.61, p = 0.44) and sibling group (F(1,27) = 1.27, p = 0.27), we observed a trend in the schizophrenia patients (F(1,26) = 3.23, p = 0.08). In which in particularly male patients showed an abnormal pattern (although the influence on social cues was also not significant for female patients).

### Social Distance Judgment Task: Klinefelter Men and Control Men

Again we first wanted to confirm the presence of an effect of social cue condition on distance judgements in the *male control group*. We performed a repeated measures ANOVA with four social cues as within-subjects factors as previously performed for the control, patient and sibling group separately. We found a significant main effect of a response bias congruent with the strength of the social cues, i.e. a significant linear increase in underestimations (i.e. increase in percentage response ‘Cartoons Closer’) of the perceived distance as strength of the social cues increased, F(1,24) = 13.54, p = 0.001. As before there was a significant difference on distance judgment between the two congruent social cue conditions A vs. D, t(24) = −3.70, p = 0.001, whereas distance judgments in the incongruent conditions, B vs. C, did not differ significantly from one another, t(24) = −0.15, p = 0.88.

In *Klinefelter men* on the other hand, the main effect of the repeated measures ANOVA was non-significant, suggesting that the percentage of response ‘Cartoons Closer’ did not change with increasing strength of the social cues, F(1,28) = 0.001, p = 0.98. Consequently, no significant difference in distance judgment was observed when comparing either the congruent social cue conditions (A vs. D), t(28) = −0.02, p = 0.98 or incongruent social cue conditions (B vs. C).

**Figure 3:**
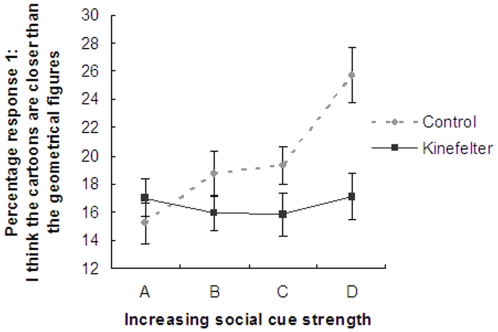
Linear increase in response 1 (“Cartoons Closer”) consistent with social cue strength in healthy control men, but not in Klinefelter men.

When comparing Klinefelter men with the control men using a repeated measures ANOVA with the four social cues as within-subject factors and Group: control men vs. Klinefelter men, as between-subjects factor, the interaction was significant, suggesting that the pattern of the sensitivity for social cues in distance estimations differed significantly between groups as reflected by different patterns of percentage response ‘Cartoons Closer’ over the four conditions, F(1,52) = 4.4, p = 0.04), which is demonstrated in Figure 3. This was specifically due to a significant difference between condition A vs. D, t(52) = −2.10, p = 0.04. Although potential age differences could have influenced the results, we did not find an effect of age on task performance, and the difference in pattern for sensitivity for social cues between the Klinefelter group and control group remained significant (p = 0.04).

### Social Distance Judgment and Symptomatology

There was a significant negative correlation between the response bias due to social cue strength and negative symptoms of schizophrenia as measured with the PANSS, Spearman's rho = −0.47, p = 0.01. This suggests that patients with more negative symptoms are less influenced by social cues. There were no significant correlations between positive symptoms or general psychopathology as measured with the PANSS and influence of social cues.

## Discussion

This study examined the implicit, or effortless processing of basic social cues, i.e. biological motion and gaze direction in three different groups: a) schizophrenia patients, b) individuals at increased genetic risk for schizophrenia, i.e. siblings of schizophrenia patients and c) individuals with an X-chromosomal disorder and high levels of schizotypal traits, i.e. men with Klinefelter syndrome.

In healthy controls, an increasing strength of social cues in the stimuli was accompanied by an increasing illusion of the perceived distance between the stimuli, indicating that social cues affected distance judgments as we expected. In contrast, in schizophrenia patients, siblings of patients and Klinefelter men, an increasing strength of social cues in the stimuli did not have any effect on the perceived distance between the stimuli, indicating that these social cues were not incorporated in the process of judging the stimuli. As a consequence these participants were less biased by social cues in their judgments and thus appear to be more accurate in their distance judgments as compared to controls. However, all groups did show a low level illusion caused by differences in mass distribution of the objects, suggesting a specific insensitivity to social cues in the experimental groups.

When considering the groups separately, schizophrenia patients and Klinefelter men were less sensitive to social cues, as compared to controls. Performance of the siblings of patients was in between patients and control participants; that is, siblings did not differ significantly from either controls or patients. The differences are probably not due to differences in general cognitive functioning, as the groups were matched on (parental) education and measures of intelligence. Furthermore, the subjects included in the analysis understood the task and were able to perform the task correctly, as indicated by their small number or absence of errors on the catch trials. In addition, the inclusion of subjects with no or very low numbers of errors on the catch trials rule out the possibility of visuospatial disabilities as well as attentional deficits that would make completion of the task difficult.

The current results suggested that patients with schizophrenia demonstrate a lack of sensitivity to even basic, simple social cues, in addition to deficits in more abstract, higher-order social cue recognition, as suggested by Corrigan and Green [10]. A failure to involuntary (implicitly) and quickly process these basic social cues may contribute to difficulties in social intuition, and hence in coping with social situations in these patients. Also, because less basic social information is available, more widespread effects on (‘upstream’-) higher-order social cognitive processing can be expected. The observed insensitivity to social cues may underlie social cognitive deficits and social dysfunction in schizophrenia. The implicit processing of social cues is thought to be especially important for the forming of a Theory of Mind, i.e. the ability to infer someone's intentions, goals and beliefs [16] and deficits in the effortless processing of these social cues might lead to disturbances in the attribution of mental states to others [14]. Indeed, a recent study demonstrated that patients with schizophrenia were impaired in using appropriate language to describe Theory of Mind animations [49].

Our results showed that especially patients with negative symptoms, which comprise social and emotional withdrawal, were insensitive to the influence of the social cues in their judgments. Patients with negative symptoms typically show problematic social functioning [50]–[52], but also deficits in other social emotional tasks [53]–[56]. Thus, these results corroborate previous research demonstrating that patients with schizophrenia show deficits in the processing of social information [6], with more severe impairments in patients with negative symptoms [56]–[59], though future research is needed to examine if any particular symptoms of the negative subscale is related to difficulties in the effortless processing of social cues. However, this study extends previous research in demonstrating deficits in the normally effortless processing of simple social cues.

Interestingly, the absence of an influence of the social cues on distance judgments was also observed in individuals at increased genetic risk for schizophrenia (siblings of patients) and in individuals with a genetic disorder associated with increased schizophrenia spectrum pathology (Klinefelter syndrome). Based on these findings three important conclusions can be drawn. First, siblings as well as the Klinefelter men were not clinically psychotic and did not use antipsychotic medication. The lack of sensitivity for social cues could thus not be due to the effects of illness or the medication use. In that way, these results validate the observed results in patients. Second, we propose that the observed lack of sensitivity for social cues is related to a genetic vulnerability to schizophrenia. The results showed that there were no differences between patients and siblings in distance judgment, suggesting that siblings resemble patients in their absence of implicit processing of social cues. However, it is important to note that the sibling group also did not differ from the control group and one could as well argue that siblings performed comparable to the control group.

Nevertheless, when taking the within group analysis into account we demonstrated that siblings, in contrast to controls, did not show a linear increase in underestimations, i.e. their distance judgments were not influenced by the social cues of human figures running towards each other or looking towards each other. Thus, our findings imply that the performance of siblings resembles the lack of sensitivity to social cues observed in schizophrenia patients, albeit to a lesser extent. Moreover, our results mirror and extend previous studies demonstrating impairments in other types of social emotional cue processing in relatives of patients with schizophrenia such as recognizing emotional facial expressions [22], [23], suggesting that problems in social cue processing might be regarded as a genetic vulnerability for schizophrenia. Third, additional evidence for a genetic loading on social cue processing comes from the finding in individuals with Klinefelter who show a similar lack of social cue processing on distance judgments as patients. As this disorder is defined by an X chromosomal abnormality, impaired cognitive processing of social cues in this group can be regarded as the expression of X-linked genetic pathology. Klinefelter men also display impairments in higher order social cognitive processing, such as recognition of facial expressions and emotional prosody, i.e. tone of voice [25]. The present findings suggest that the insensitivity to social cues could be regarded as an endophenotype that is shared by schizophrenia patients and Klinefelter men. Hence, not only in Klinefelter syndrome, but also in the schizophrenia spectrum, we might consider a role of X-linked genetic pathology underlying impairments in effortless processing of social information. This might explain, at least in part, the sex differences that have been observed in the incidence and severity in schizophrenia [34], [60], although we only observed a trend for male schizophrenia patients to be less sensitive to social cues compared to female schizophrenia patients. Moreover, this cognitive endophenotype may may also be present in other psychiatric disorders characterized by social cognitive deficits and the recognition of endophenotypes can contribute to the early detection of and possibly preventive treatment for certain psychiatric disorders.

With regard to the neural correlates involved in the processing of biological motion and social attention, the superior temporal gyrus, medial prefrontal cortex and anterior cingulate have been implicated [15]. Both in schizophrenia patients as well as relatives, abnormalities in these regions have been reported [61]–[67]. Interestingly, structural abnormalities in the anterior cingulate and the superior temporal gyrus have been found in Klinefelter syndrome as well [68]. Future studies should relate neural substrates of social cue processing in schizophrenia and relatives together with measures of social functioning. This would elucidate the relationship between the ability to process social cues and social behaviour and its underlying brain pathology in schizophrenia and provide more insight into the biological vulnerability to schizophrenia.

Finally, it is important to note some limitations of this study. For instance, it would be interesting to include a patient control group that is not associated with an increased risk to develop schizophrenia to demonstrate that this patient group is indeed susceptible to the illusion. In addition, a non-social condition could be included in the task to substantiate that the absence of the illusion is specific for social cues. Especially because previous research on visual illusions in schizophrenia has shown a reduced susceptibility in schizophrenia [69]–[71] and thus the current results need to be interpreted with caution. Nevertheless, we did observe the presence of a low-level illusion in all experimental groups demonstrating that patients with schizophrenia, their siblings and XXY men are susceptible to some perceptual illusions unrelated to these social cues. Further this highlights that other cognitive deficits, such as working memory, attention or visual deficits, probably do not explain our results. Another issue concerns a possible selection bias in the Klinefelter group. Since many men with Klinefelter syndrome remain undiagnosed [72] and untreated, the present results might not generalize to the general Klinefelter population. Finally our results might have been different if we had used more realistic stimuli in which decoding of social cues is more relevant instead of cartoon figures.

In summary, this study investigated the influence of simple, usually implicitly processed, basic social cues, i.e. biological motion and gaze direction, on distance judgements in individuals with a) a diagnosis of schizophrenia b) an increased risk for schizophrenia (siblings of patients) and c) with a genetic disorder associated with increased schizophrenia spectrum pathology (Klinefelter syndrome). Results showed that patients with schizophrenia, siblings of patients with schizophrenia and Klinefelter men (47, XXY) did not process these social cues effortlessly (involuntary or implicitly) compared to healthy controls. Within the schizophrenia group, this was especially the case in patients with more severe negative symptoms, i.e. patients that show additional social emotional disturbances. Hence, social cue processing deficits seem related to the vulnerability for schizophrenia, instead of illness in general and with a potential involvement of genes on the X chromosome. These basic social cue processing deficits might underlie impairments in other aspects of social cognition and social functioning. Future research should investigate the relationships among insensitivity to social cues, social functioning and neurobiological substrates in schizophrenia as well as schizotypal symptoms in high-risk groups.
